# Supplementary material for: Screening of Lactiplantibacillus plantarum with High Stress Tolerance and High Esterase Activity and Their Effect on Promoting Protein Metabolism and Flavor Formation in Suanzhayu, a Chinese Fermented Fish
Source: Foods. 2022 Jun 29;11(13):1932. doi: 10.3390/foods11131932 (PMC9265898; doi:10.3390/foods11131932)
Supplement: Supplementary file 1 [file foods-11-01932-s001.zip › foods-1769206-supplementary.pdf]

Supplementary Materials

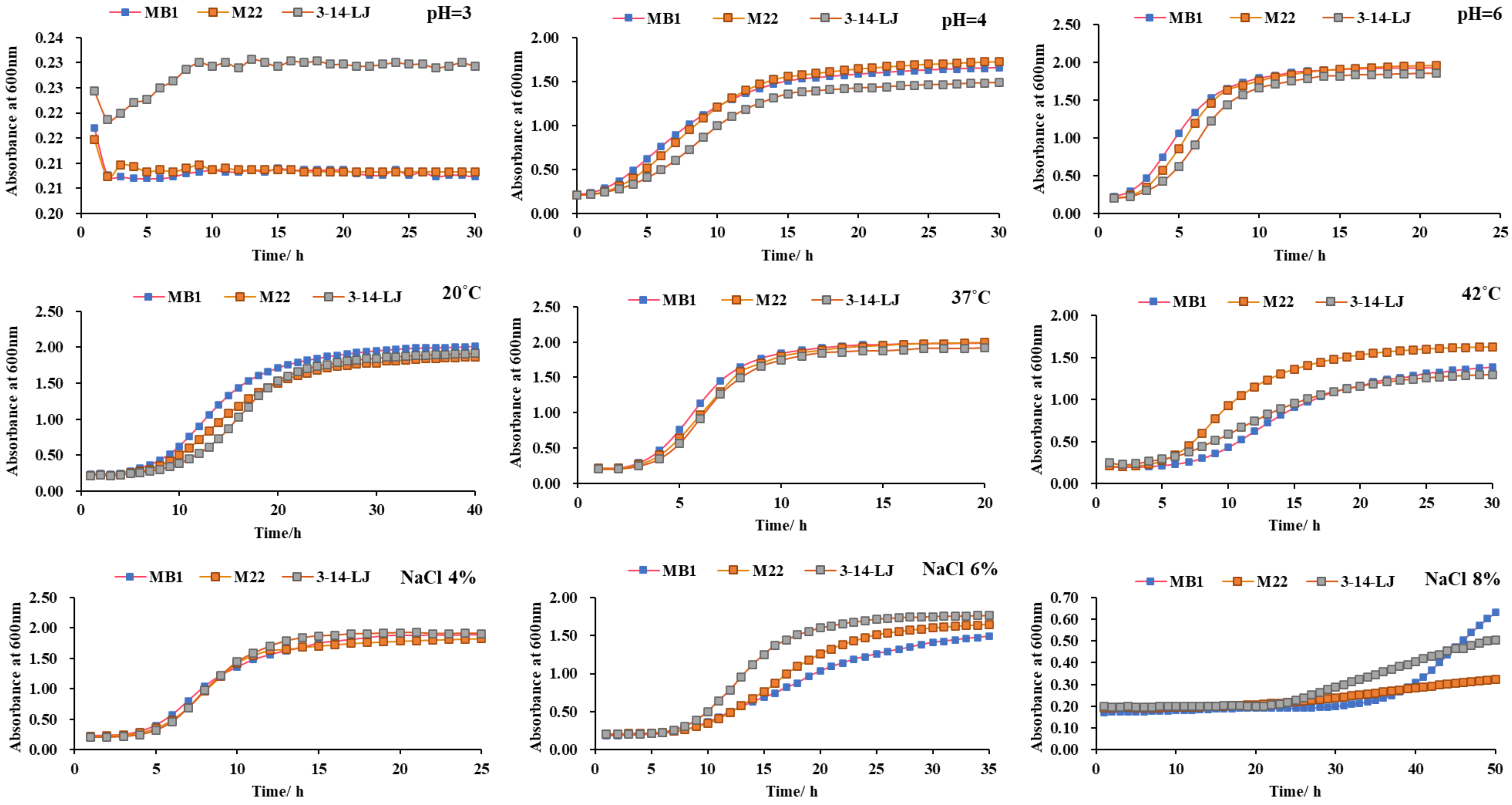

Figure S1 Growth characteristics of *L. plantarum* M22, MB1 and 3-14-LJ in different pH, temperature and NaCl concentrations.

Table S1 Contents of volatile compounds in model system of *Suanzhayu*.

	Content (μg/100 g)					Identification method
	MF	MC	M3	M2	M1	
**alcohols**						
2-ethyl-1-hexanol	120.57±8.97	30.36±6.06	42.31±5.81	193.01±21.81	78.47±16.68	MS/RI
1-eexanol	62.10±7.93	109.86±14.16	417.53±55.25	286.68±17.5	316.90±18.76	MS/RI
5-octen-1-ol	3.3±0.41	5.63±0.71	6.78±2.75	15.94±1	14.46±2.65	MS/RI
1-octanol	6.34±1.06	8.36±0.93	25.30±16.65	55.83±1.71	70.34±8.05	MS/RI
1-nonanol	3.76±0.17	10.55±1.85	24.33±17.09	40.59±4.37	67.53±15.14	MS/RI
1-octen-3-ol	45.75±3.71	95.05±13.10	97.70±10.12	125.82±21.57	119.01±6.53	MS/RI
2-butyl-1-octanol	ND	ND	2.56±2.30	ND	ND	
subtotal	241.85±6.64[c]	259.80±12.38[c]	616.52±17.70[b]	717.87±64.94[a]	666.71±67.27[ab]	
**aldehydes**						
hexanal	118.58±15.7	347.03±38.97	292.09±10.6	433.51±52.05	292.7±18.73	MS/RI
octanal	12.25±1.66	9.73±1.59	28.43±6.67	38.83±10.17	37.34±4.65	MS/RI
nonanal	48.18±4.9	35.18±3.68	58.72±7.08	110.38±4.49	84.36±7.55	MS/RI
heptanal	6.88±0.88	ND	9.77±6.15	10.83±1.86	8.76±1.87	MS/RI
3-butylacrolein	3.25±0.52	20.22±0.86	21.43±1.17	25.24±0.55	23.3±0.76	MS/RI
(e)-2-nonenal	2.23±0.21	8.96±1.14	14.58±3.99	33.77±0.95	31.75±1.79	MS/RI
2,4-noadienal	ND	ND	4.81±1.82	ND	ND	MS/RI
2,5-dimethyl-benzaldehyde	ND	ND	ND	65.95±1.61	ND	MS/RI
(e, e)-2,4-decadienal	ND	4.74±1.68	5.8±0.83	11.8±0.63	11.88±1.77	MS/RI
2-undecenal	ND	ND	7.39±3.83	11.79±1.22	12.86±2.24	MS/RI
2,4-dimethyl-benzaldehyde	ND	ND	ND	ND	33.53±4.7	MS/RI
(e)-2-decenal	ND	3.13±0.88	12.58±4.74	18.95±0.89	25.77±2.05	MS/RI
subtotal	191.39±21.63[d]	428.99±44.68[c]	445.62±19.49[c]	761.07±66.00[a]	562.22±28.25[b]	
**esters**						
formic acid hept-2-yl ester	4.19±0.28	6.64±0.17	16.65±6.03	23.58±2.63	31.77±2.68	MS/RI
allyl 2-ethyl butytate	17.93±2.36	12.42±5.49	ND	ND	ND	MS/RI
isoamyl acetate	ND	ND	6.05±0.31	8.82±0.27	4.38±0.26	MS/RI/S
ethyl hexanoate	ND	ND	1.85±0.23	2.17±0.25	ND	MS/RI/S
ethyl octanoate	ND	ND	1.44±0.09	1.72±0.13	1.48±0.06	MS/RI/S
subtotal	22.12±2.59[b]	19.50±5.61[b]	26.00±6.50[b]	36.29±2.55[a]	37.63±2.83[a]	

**ketones**						
3,5,5-trimethyl-2-cyclohexen-1-one	ND	ND	ND	8.45±0.59	4.83±1.35	MS/RI
subtotal	ND	ND	ND	8.45±0.59[a]	4.83±1.35[b]	
**alkanes**						
2,6,11-trimethyldidecane	ND	ND	3.40±1.51	ND	ND	MS/RI
pentadecane	ND	ND	ND	4.38±0.91	4.57±1.37	MS/RI
2-methyldecane	ND	3.00±2.22	ND	6.55±0.21	3.32±0.73	MS/RI
subtotal	ND	3.00±2.22[c]	3.40±1.51[c]	10.93±0.94[a]	7.89±2.07[b]	
**acids**						
palmitic acid	ND	ND	ND	24.53±14.79	ND	MS/RI
oleic acid	ND	ND	ND	12.87±8.13	ND	MS/RI
subtotal	ND	ND	ND	37.40±22.56[a]	ND	
**others**						
2,5-dimethylcyclohexanol	ND	15.78±1.12	9.22±1.84	12.37±0.87	16.92±4.16	MS/RI
1,3-ditertiarybutylbenzene	ND	19.29±1.14	25.32±8.87	ND	ND	MS/RI
2-pentylfuran	14.22±0.80	15.56±2.68	45.93±10.64	115.18±6.64	98.57±9.68	MS/RI
2,6-dimethylonane	5.88±0.50	ND	4.21±1.31	ND	ND	MS/RI
subtotal	20.1±1.27[a]	50.63±2.38[c]	84.67±19.89[b]	127.55±6.96[a]	115.50±5.53[a]	
**total**	475.49±31.71[e]	761.48±25.09[d]	1186.21±51.83[c]	1691.12±152.16 aa	1389.99±101.16[b]	

"ND": not detected. Abbreviations see Table 1. Letters "a-e" indicate the significant difference (*P* <0.05).

Identification based on Nist 11 mass spectral database; published retention indices; authentic standards

Table S2 Contents of volatile compounds in actual system of *Suanzhayu.*

	Content (mg/100g)					Identification method
	AF	AC	A3	A2	A1	
**alcohols**						
1-pentanol	ND	ND	202.58±97.51	ND	ND	MS/RI
2,3-butanediol	ND	ND	64.83±36.29	769±10.25	334.59±175.25	MS/RI
2,2-dimethyl-1-butanol	ND	ND	ND	94.09±86.23	ND	MS/RI
2-hexanol	115.98±90.88	ND	ND	1329.76±1201.64	588.85±337	MS/RI
1-hexanol	ND	114.64±82.29	ND	151.83±82.86	87.81±21.28	MS/RI
3-methyl-2-hexanol,	ND	ND	68.76±53.1	ND	112.72±46.35	MS/RI
(e)-2-octenal	ND	ND	55.78±3.77	42.32±16.73	73.96±38.98	MS/RI
1-octen-3-ol	82.19±14.46	322.58±23.69	445.5±189.41	243.33±170.56	425.78±77.86	MS/RI
3-octanol	ND	63.06±22.98	53.04±17	58.72±57.94	ND	MS/RI
2-ethyl-1-hexanol	244.34±92.24	861.69±248.82	552.58±118.29	1186.8±349.88	756.97±150.47	MS/RI
subtotal	442.51±135.53[c]	1361.96±331.82[bc]	1240.48±352.34[bc]	3708.76±1218.53[a]	2380.68±671.46[b]	
**alkanes**						
3,5,5-trimethyl-1-hexene	ND	184.73±10.91	ND	ND	ND	MS/RI
2-methyl-1-octene	62.12±40.24	ND	308.38±58.77	ND	118.83±95.24	MS/RI
2,2-dimethyl-heptane	ND	282.67±203.09	1374.89±317.59	ND	199.57±74.68	MS/RI
hexadecanal	ND	ND	33.05±8.68	47.64±20.88	ND	MS/RI
subtotal	62.12±40.24[c]	467.4±214[b]	1716.33±318.05[a]	47.64±20.88[c]	318.4±165.99[bc]	
**esters**						
2-methoxy acetate	ND	ND	259.53±327.25	522.63±260.3	ND	MS/RI
isobutyl acetate	ND	78.02±48.11	ND	73.33±16.88	ND	MS/RI
2-methyl-propanoic acid ethyl ester	ND	ND	480.29±455.8	225.32±168.58	ND	MS/RI
methyl 2-methylbutyrate	ND	ND	ND	232.94±284.66	ND	MS/RI
l-lactic acid ethyl ester	ND	1295.23±823.3	411.82±256.14	859.81±414.2	ND	MS/RI
2-methoxy ethyl acetate	ND	572.28±313.36	75.82±87.39	415.33±613.92	530.31±79.42	MS/RI
butyl acetate	ND	164.06±43.89	ND	96.14±83.75	682.59±698.3	MS/RI
2-ethylhexyl acetate	ND	126.64±96.22	25.78±7.99	42.22±6.64	ND	MS/RI
hexyl acetate	ND	ND	25.01±4.19	ND	ND	MS/RI
3-methylbutyl 2-methylprop-2-enoate	ND	119.75±62.25	52.18±35.65	60.2±29.37	128.03±145.43	MS/RI
formic acid 1-propen-2-yl ester	ND	ND	33.33±8.67	ND	48.23±33.8	MS/RI

fumaric acid decyl 3-oxobut-2-yl ester	ND	ND	ND	34.87±13.96	ND	MS/RI
2-methylpentyl formate	ND	ND	318.04±279.84	88.61±94.07	ND	MS/RI
allyl butyrate	105.63±31.83	ND	ND	373.95±106.24	345.98±119.34	MS/RI
isoamyl acetate	ND	271.08±4.15	117.55±5.75	205.05±0.39	64.64±0.23	MS/RI/S
ethyl caproate	ND	191.27±088	61.55±0.48	68.26±0.37	47.10±0.19	MS/RI/S
ethyl octanoate	ND	ND	36.58±0.61	67.39±0.78	13.33±0.26	MS/RI/S
subtotal	105.63±31.83^{b}	2818.33±781.56^{a}	1938.83±847.51^{a}	3366.07±1087.28^{a}	1860.21±716.21^{a}	
**aldehydes**						
hexanal	741.7±220.8	709.66±394.29	1602.57±294.17	ND	ND	MS/RI
benzaldehyde	ND	ND	36.52±13.64	45.17±14.69	ND	MS/RI
octanal	37.32±15.06	48.37±12.28	168.96±37.52	65.18±33.46	ND	MS/RI
nonanal	147.72±41.2	112.07±9.65	576.16±173.99	395.83±112.27	595.62±90.29	MS/RI
subtotal	926.75±169.16^{b}	870.1±391.66^{b}	2384.21±380.23^{a}	506.17±155.74^{b}	595.62±90.29^{b}	
**ketones**						
3-hexanone	ND	124.84±45.24	ND	ND	ND	MS/RI
3-octanone	ND	ND	260.04±17.56	ND	ND	MS/RI
subtotal	ND	124.84±45.24^{b}	260.04±17.56^{a}	ND	ND	
**acids**						
palmitic acid	128.54±124.96	38.69±9.53	ND	1034.72±502.13	ND	MS/RI
oleic acid	ND	ND	ND	236.06±237.07	ND	MS/RI
subtotal	128.54±124.96^{b}	38.69±9.53^{b}	ND	1270.78±658.20^{a}	ND	
**others**						
2-(2-pentenyl) furan	ND	40.8±1.71	41±3.38	ND	ND	MS/RI
2-pentyl- furan	24.05±14.26	123.02±43.24	447.7±175.86	110.9±80.33	468.72±120.19	MS/RI
diethyl-cyanamid	68.31±77.65	ND	ND	ND	ND	MS/RI
acetoin	ND	ND	2071.17±409.1	1921.49±493.35	1632.42±825.95	MS/RI
3-methyl-butanoic acid	ND	35.77±2.69	62.77±14.11	308.95±402.59	109.91±51	MS/RI
1-chloro-octane	ND	ND	38.33±9.56	ND	ND	MS/RI
subtotal	92.36±91.72^{b}	199.60±42.26^{b}	2660.97±567.16^{a}	2341.34±755.01^{a}	2211.05±756.37^{a}	
**total**	1757.91±101.08^{c}	5880.93±1387.56^{b}	10403.45±917.18^{a}	11240.77±1706.70^{a}	7365.95±417.95^{b}	

“ND”: not detected. Abbreviations see Table 1. Letters “a-d” indicate the significant difference (P <0.05).

Identification based on Nist 11 mass spectral database; published retention indices; authentic standards
